# Reference Values for Water‐Specific T1, Intermuscular and Intramuscular Fat Content in Skeletal Muscle at 2.89 T

**DOI:** 10.1002/jmri.29718

**Published:** 2025-01-24

**Authors:** Stephen J. Foulkes, Mark J. Haykowsky, Rachel Sherrington, Amy A. Kirkham, Justin Grenier, Peter Seres, David I. Paterson, Richard B. Thompson

**Affiliations:** ^1^ Integrated Cardiovascular Exercise Physiology and Rehabilitation Lab, College of Health Sciences, Faculty of Nursing University of Alberta Edmonton Alberta Canada; ^2^ Heart, Exercise and Research Trials Lab St Vincent's Institute of Medical Research Melbourne Victoria Australia; ^3^ Department of Biomedical Engineering University of Alberta Edmonton Alberta Canada; ^4^ Faculty of Kinesiology & Physical Education University of Toronto Toronto Ontario Canada; ^5^ Department of Radiology and Diagnostic Imaging University of Alberta Edmonton Alberta Canada; ^6^ Division of Cardiology University of Ottawa Heart Institute Ottawa Ontario Canada

**Keywords:** SR‐CSE, MRI, skeletal muscle, T1 mapping, MOLLI, SASHA, PDFF

## Abstract

**Background:**

MRI offers quantification of proton density fat fraction (PDFF) and tissue characteristics with T1 mapping. The influence of age, sex, and the potential confounding effects of fat on T1 values in skeletal muscle in healthy adults are insufficiently known.

**Purpose:**

To determine the accuracy and repeatability of a saturation‐recovery chemical‐shift encoded multiparametric approach (SR‐CSE) for quantification of T1_Water_ and muscle fat content, and establish normative values (age, sex) from a healthy cohort.

**Study Type:**

Prospective observational; phantoms (NiCL_2_‐agarose T1 phantoms with no fat content; gadolinium T1 phantoms with mixed fat‐water content).

**Populations:**

A total of 130 healthy community‐dwelling adults (63 male, 18–76 years) free of chronic health conditions that require regular prescription medication, and with no contraindications to MRI.

**Field Strength/Sequence:**

2.89 T; gradient echo sequences including saturation‐recovery chemical‐shift encoded T1 mapping (SR‐CSE); MOLLI; SASHA; CSE; and single voxel spectroscopy.

**Assessment:**

SR‐CSE provided T1_Water_ and PDFF maps for assessment of intramuscular (MF_Intra_), intermuscular (MF_Inter_), and subcutaneous fat and muscle volumes (thigh, paraspinal muscles). Comparison with MOLLI/SASHA T1 mapping.

**Statistical Tests:**

Univariable and multivariable linear regression, general linear models, Bland and Altman, coefficient of variation (CV). *P*‐value <0.05 was considered statistically significant.

**Results:**

Phantom and in vivo validation studies showed excellent accuracy of SR‐CSE T1_Water_ and PDFF vs. values from reference standards and repeatability CVs between 0.2% and 2.6% for T1_Water_, R2*, MF_Inter_, MF_Intra_, subcutaneous fat and muscle volumes. Mean T1_Water_ was 36 msec significantly higher in females (1445 ± 23 msec vs. 1409 ± 22 msec), with no age‐effect (*P* = 0.35). Females had significantly higher values for MF_Inter_ (10.4% ± 4.8% vs. 7.1% ± 2.9%) and MF_Intra_ (2.6% ± 1.0% vs. 2.3% ± 0.8%), both of which increased with age, secondary to lower muscle volume. MOLLI and SASHA T1 values had a fat‐related bias of 21.7/35.0 msec per 1% increase in fat fraction (MFF_Intra_), in vivo, and a constant bias of −319.8/+35.6 msec, respectively.

**Data Conclusion:**

SR‐CSE provides accurate (vs. phantoms) and repeatable assessment of water‐specific T1 values and muscle and fat volumes. Conventional methods (SASHA, MOLLI) have a significant fat‐modulated T1‐bias. T1_Water_ values are higher in females with no significant age dependence.

**Plain Language Summary:**

We developed and tested the accuracy of a new MRI approach to measure tissue damage in skeletal muscle using a method called T1 mapping. The approach also provided matching images of fat within the muscle. We measured T1 values and muscle fat volumes in the thighs of 130 healthy adults to define normal values in healthy people and to understand if these values are influenced by age, sex, or weight. We found that our MRI technique accurately measured T1 values and fat volumes within muscle and we defined normal ranges of values, which were different in healthy males and females.

**Level of Evidence:**

2

**Technical Efficacy:**

Stage 1

MRI offers a noninvasive, safe, and reproducible technique to evaluate skeletal muscle disorders.^1^ MRI can differentiate fat and water signals to identify disorders associated with the burden and pattern of intermuscular fat (MF_Inter_), intramuscular fat (MF_Intra_), and changes in muscle microstructure and water content (fibrosis, edema, and inflammation).^1^, ^2^ Skeletal muscle fat is increasingly utilized as a mechanistic, diagnostic, and therapeutic target in aging, neuromuscular disorders, orthopedics, cancer, heart failure, and diabetes^2^, ^3^ based on its association with metabolic and cardiovascular health,^4^, ^5^, ^6^, ^7^ physical performance,^2^, ^5^, ^8^, ^9^ and muscle wasting.^2^ Edema, inflammation, and fibrosis are also considered critical markers of disease activity (or therapeutic efficacy) in skeletal muscle.^2^ However, there is no standardized approach to assess these complimentary outcomes or consensus normative values.

Typically, MF_Inter_ refers to the bulk fat pool between the muscle groups while MF_Intra_ refers to the fat between and within the muscle fibers, although there are no universal definitions.^3^, ^10^, ^11^ These fat pools have distinct morphology and biology, and may have differential pathological implications,^3^ so would ideally be distinguished in assessment of muscle fat content. Furthermore, the presence of MF_Intra_ within the muscle region (i.e., within image voxels that are predominantly water) may also confound complimentary muscle‐specific metrics derived from the water pool within the muscle, in particular, T1 mapping for evaluation of fibrosis and edema.^12^, ^13^, ^14^ It has been shown that the presence of fat in liver and skeletal muscle may lead to large potential bias in T1 values measured with commonly used T1 mapping methods.^9^, ^12^, ^15^, ^16^, ^17^, ^18^ This is an important consideration for interpreting T1 values in skeletal muscle, as the targeted tissue disorders (edema, inflammation, fibrosis) often coexist with increased MF_Intra_.^2^, ^3^

Emerging composite imaging methods enable simultaneous quantification of PDFF and water‐specific T1 (T1_Water_).^14^, ^17^, ^19^, ^20^, ^21^ These methods address fat‐related T1 biases by separating water and fat signals in conjunction with T1 mapping to yield T1_Water_ values with simultaneous fat fraction imaging. The purpose of the current study was to determine normative values for T1_Water_ in skeletal muscle in healthy adults, with consideration of age and sex. A secondary goal was to measure normative skeletal muscle fat fractions (%), MFF_Inter_ and MFF_Intra_, and fat volumes (mL), MFV_Inter_ and MFV_Intra_, also with consideration of age and sex. The fat‐modulated T1 bias in skeletal muscle without water‐specific mapping was also evaluated using SASHA^22^ and MOLLI^23^ T1 mapping acquisitions to determine T1 bias in skeletal muscle as a function of muscle fat content for these commonly used methods.

## Materials and Methods

### Study Participants

This study was approved by the institutional ethics board and written informed consent was obtained from all study participants. A cohort of 130 healthy participants (≥18 years of age, 63 males, 67 females) were prospectively recruited from a single site to derive normative MRI data. Healthy adults were defined as (and considered for inclusion) if they had no diagnosed diseases or conditions requiring regular medication use, and had no MRI contraindications. A subset of two participants underwent additional studies for in vivo spectroscopic validation (covering 14 regions of interest), 20 participants underwent assessments for repeatability of T1_Water_ and MF_Inter_ and MF_Intra_, and nine participants underwent additional comparison to T1 mapping (covering 277 regions of interest) with widely available methods without water‐specific evaluation, as described below.

### Image Acquisition

All imaging studies were performed on a 2.89 T MRI scanner (MAGNETOM Prisma; Siemens Healthcare; Erlangen, Germany) using the spine and body matrix radiofrequency coils for signal reception for phantoms, from both legs, or from paraspinal muscles.

Phantom studies (NiCL_2_‐agarose T1 phantoms with no fat content; gadolinium T1 phantoms with mixed fat‐water content) used for validation of the SR‐CSE sequence are detailed in Section S1 in the Supplemental Material, including pulse sequence parameters and phantom design.

#### IN VIVO STUDIES

**FIGURE 1:**
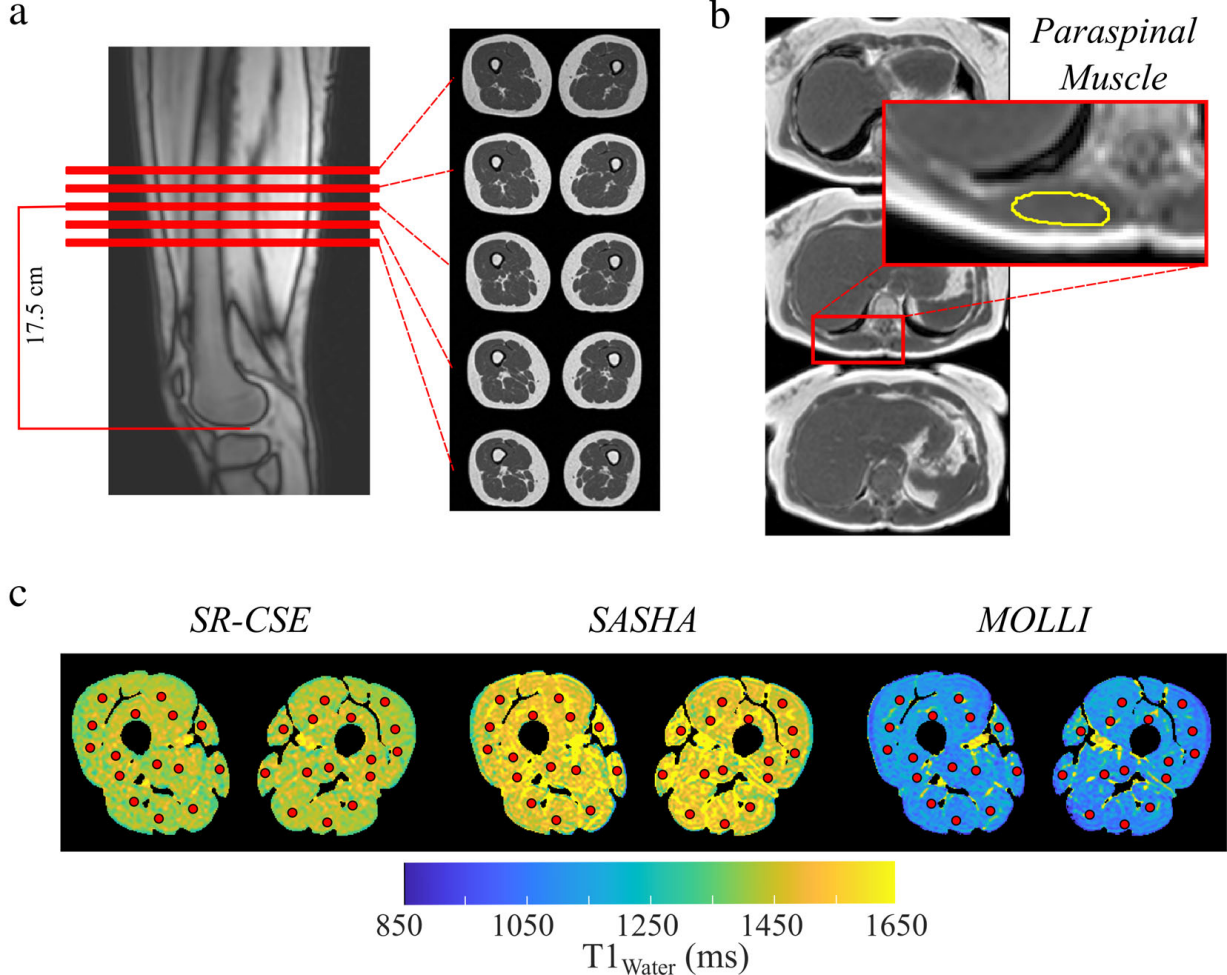
Thigh and paraspinal muscle slice prescriptions. (**a**) Standardized thigh slice locations for all in vivo studies (five slices). (**b**) Paraspinal muscle slice locations (three slices). (**c**) Illustrative locations of 30 regions of interest on SR‐CSE, SASHA, and MOLLI T1 maps used for method comparison.

- *SR‐CSE*—A variation of the previously reported SR‐CSE gradient echo approach^17^ was used for all thigh imaging studies in 130 healthy participants and an illustrative clinical case with type 2 diabetes. The SR‐CSE sequence was prescribed in a transverse slice orientation covering both thighs with the central slice 17.5 cm above the distal point of the femur to provide a reproducible mid‐thigh location (Fig. 1a). All healthy participants also underwent SR‐CSE scans of the liver as part of a separate published study,^24^ which provided opportunistic coverage of the paraspinal muscles to enable comparison of thigh muscle and paraspinal muscle T1_Water_ and MFF_Intra_ values (Fig. 1b). SR‐CSE thigh acquisitions were repeated in 20 participants for determination of scan‐rescan repeatability of T1_Water_, R2*, and muscle composition.
- *SASHA* ^22^ and *MOLLI* ^23^ *gradient echo T1 mapping*—T1 maps were acquired in a subset of nine healthy participants (thigh) for comparison with SR‐CSE T1_Water_ values, with slice locations matching the central SR‐CSE slice (Fig. 1c).
- *Conventional gradient echo CSE*—Images were acquired in a subset of 41 healthy participants (thigh) for comparison with SR‐CSE MF_Inter_ and MF_Intra_ values, with matching slice locations for all five slices.
- *Single Voxel* ^*1*^ *H Spectroscopy*—Spectroscopy studies were completed in 14 discrete regions from two healthy participants (thigh) from several thigh muscle groups for in vivo validation of SR‐CSE MFF_Intra_ values. Single voxel spectroscopy acquisitions from intramuscular regions using the STEAM approach were prescribed on the central slice from SR‐CSE acquisitions, taking care to avoid MF_Inter_ fat pools between muscle groups.

#### PULSE SEQUENCE

The SR‐CSE pulse sequence employs a combination of multi‐echo acquisitions, to enable CSE for fat and water separation and R2* mapping, and saturation recovery preparations for T1_Water_ quantification from the water‐separated images.^17^ Water‐separated images, with and without saturation‐recovery preparation, are subsequently used to calculate T1_water_ values (Fig. S2A in the Supplemental Material). Pulse sequence parameters used for all phantom and in vivo studies of the thigh included: 450 mm × 225 mm FOV, 3.5 mm slice thickness with a 10.5 mm gap, 5 slices, 224 × 112 acquisition matrix and GRAPPA = 2 (external lines) for 56 total acquired k‐space lines. The five axial slices covered 59.5 mm centered 17.5 cm above the distal point of the femur (Fig. S3A in the Supplemental Material). A dual‐echo acquisition with monopolar readouts was repeated three times with a 1.14 msec increment in the echo time for a total of six echo times with five unique TE times, TE1 = 2.51, 3.65, and 4.79 msec and TE2 = 4.79, 5.93, and 7.07 msec, all with a constant TR = 10.98 msec. The echo‐shifting approach^25^ enabled reduced inter‐echo spacing of 1.14 msec in conjunction with a relatively low receiver bandwidth of 795 Hz/pixel for increased SNR. All image acquisitions were single‐shot with slice‐interleaving and a linear k‐space trajectory. As previously detailed, minimal T1‐weighting was designed with the use of a sin^3^(*θ*) flip angle pattern of 0 < *θ* < *π*/2 over k‐space with a flip angle of 30° at the center of k‐space.^17^ Acquisitions were repeated with a reversal of the phase‐encoding ordering and then averaged in k‐space to ensure uniform signal yield across k‐space and to increase signal to noise ratios from the averaging of the paired acquisitions.^17^ Acquisitions were also repeated with and without saturation‐recovery preparation for T1 quantification, with a saturation recovery time at the middle of k‐space of 1537.2 msec for all acquisitions. The total image acquisition time was 77 seconds for the five slices. Paraspinal SR‐CSE images were acquired with a faster 10‐second breath‐hold duration pulse sequence variant that was previously described and validated.^17^, ^24^ Three transverse slices were prescribed primarily for liver imaging with opportunistic coverage of the erector spinae muscle groups (Fig. 1b).

Single slice 5(3)3 MOLLI T1 acquisitions were prescribed on the central SR‐CSE slice location in the thigh with an FOV of 450 × 225 mm^2^, slice thickness of 3.5 mm, 224 × 112 matrix, GRAPPA = 2 (external lines), a receiver bandwidth of 1395 Hz/pixel, TE = 1.23 msec, and a 35° flip angle. Matching single slice SASHA T1 acquisitions used identical parameters but with a 70° flip angle. A synthetic ECG signal with an R‐R interval of 1 second was used for all MOLLI and SASHA acquisitions.

A conventional multi‐echo gradient echo pulse sequence used slice thickness and locations to match SR‐CSE acquisitions. Pulse sequence parameters included a 470 mm × 239 mm FOV, 3.5 mm slice thickness with a 10.5 mm gap, five slices with slice interleaving, 256 × 130 acquisition matrix and GRAPPA = 2 (external lines), TE = 2.04, 3.74, 5.57, 7.53, 9.49, and 11.45 msec, TR = 20 msec and a 20° flip angle. A fat and water T1‐correction based on calculated signal yields using the spoiled gradient echo contrast equation was applied during image processing to account for higher signal yield for fat compared to water in skeletal muscle.^26^

Single voxel spectroscopy for measurement of MF_Intra_ used the STEAM approach with TE = 15 msec, TM = 10 msec, TR = 10 seconds, 1300 Hz/pixel bandwidth, 16 averages, 0.35 cm × 1 cm × 1 cm voxel volume with no fat suppression. The fat and water areas were corrected for T2 signal loss using STEAM acquisitions with TE = 15 , 30, and 50 msec.

### Image Reconstruction and Image Processing

The processing pipeline for the calculation of T1_Water_, PDFF, and T2* maps for the SR‐CSE method have been described and validated (Fig. S2B in the Supplemental Material), including illustration of the insensitivity of all calculated values to B_1_^+^ inhomogeneity and off‐resonance effects.^17^

Fat and water separated images were calculated separately for saturation‐recovery and nonsaturation‐recovery images using a graph cut algorithm.^27^ A six‐peak fat model used peak locations (relative to water) at 3.73, 3.33, 2.52, 1.93, 0.383, and − 0.67 ppm with relative weights of 0.0870, 0.693, 0.1280, 0.0040, 0.0390, and 0.0480, respectively.^28^ Magnetic field and R2* maps were calculated as part of the fat‐water separation process and all calculations included R2* correction. These maps were jointly calculated for saturation‐recovery and nonsaturation‐recovery images, ensuring identical magnetic field and R2* maps, but unique fat and water separated images. The same algorithm and fat model was used for fat and water separation for conventional CSE acquisitions.

Values for T1_Water_ were calculated from saturation and nonsaturation recovery images (Fig. S2B in the Supplemental Material) using a lookup table approach based on Bloch equation simulations of the complete pulse sequence to account for pulse sequence effects on T1 quantification.^17^

A custom machine learning segmentation model targeted muscle, subcutaneous fat, intermuscular fat, skin and bone regions in the thigh. Development and validation of the machine learning segmentation model is detailed in Section S3 in the Supplemental Material. Reported values for T1_Water_, PDFF and R2* were calculated in the segmented muscle pixels, where the PDFF in this pool was defined as MFF_Intra_. In vivo values for T1_Water_, MFF_Intra_, and R2* were calculated as mean values from all segmented muscle tissue pixels from all five slices. Calculation methods for fat fractions and volumes are detailed in Section S3 in the Supplemental Material. Muscle and fat volumes were corrected to include the slice gaps to represent the total slab thickness of 59.5 mm. The same processing approach was applied to conventional CSE fat and water separated images.

For comparison of SR‐CSE with MOLLI and SASHA T1 maps, 30 circular regions of interest with a radius of 0.5 mm (area of 0.785 mm^2^) were selected by a single investigator (R.B.T.) over both thighs for each of the nine comparison datasets (Fig. 1c). The selected regions avoided intermuscular fat and/or blood vessels.

### Statistical Analysis

Data was analyzed using IBM SPSS Statistics (version 29.0.0.0, IBM Corp). Continuous descriptive data are reported as mean ± standard deviation (SD) or (range), whilst categorical data are reported as frequencies (percentage). The agreement between SR‐CSE derived metrics and the referent standards were compared using Bland and Altman plots and linear regression. Repeatability of SR‐CSE‐derived measures of muscle composition was determined using coefficient of variation (CV) and Bland and Altman plots. Normative values for muscle composition were aggregated by sex, and reported as mean ± SD and 5th and 95th percentiles. The effect of age and sex on muscle composition and T1_Water_ was assessed using multivariable regression. Body mass index was not included in this model due to collinearity with all fat volumes and percentages. Instead, the impact of BMI on muscle composition and T1_Water_ values was assessed using general linear models (with sex and BMI ≥25 kg/m^2^ included as fixed factors) with Bonferroni correction for multiple comparisons. Statistical significance was set at *P* < 0.05 for all analyses.

## Results

### Phantoms

**FIGURE 2:**
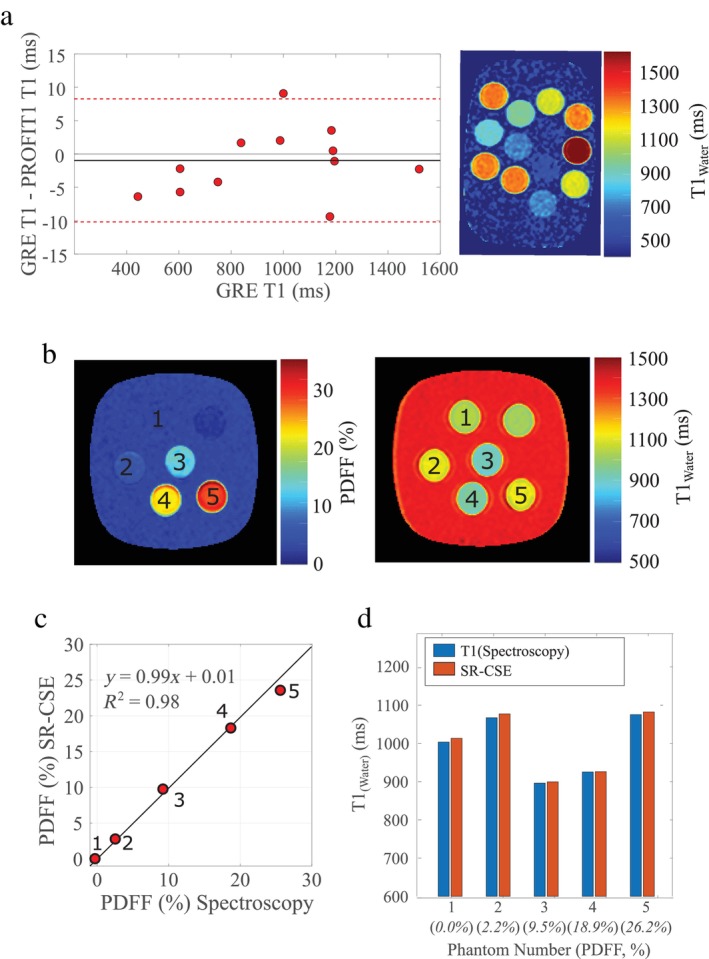
Phantom validation of SR‐CSE T1_Water_ and PDFF. (**a**) Bland–Altman plot of SR‐CSE T1 values vs. gradient‐echo T1 measurement (GRE) in NiCl_2_ phantoms (water phantoms). The SR‐CSE T1_Water_ map is shown. (**b**) SR‐CSE proton‐density fat fraction (PDFF) and T1_Water_ images of agar fat‐water phantoms with increasing fat content. (**c**) Comparison of PDFF assessed by SR‐CSE and ^1^H spectroscopy in the five tubes from (b). (**d**) Comparison of SR‐CSE T1_Water_ values in the five tubes with increasing PDFF from (b) with spectroscopic T1 evaluation.

There was excellent agreement between SR‐CSE T1_Water_ and saturation‐recovery gradient‐echo T1 values in NiCl_2_ water‐only phantoms (Fig. 2a; <1 msec bias; 95% limits of agreement ~10 msec). SR‐CSE T1_water_ values also showed good agreement with saturation‐recovery single‐voxel spectroscopy in mixed fat‐water phantoms (Fig. 2d; −6 msec bias; 95% limits of agreement ~8 msec). PDFF values measured by SR‐CSE also showed very close agreement with ^1^H spectroscopy (Fig. 2c; *R* ^2^ = 0.98). The five sample tubes are labeled in the SR‐CSE PDFF and T1_Water_ images from the mixed fat and water phantoms (Fig. 2b).

### In Vivo

**FIGURE 3:**
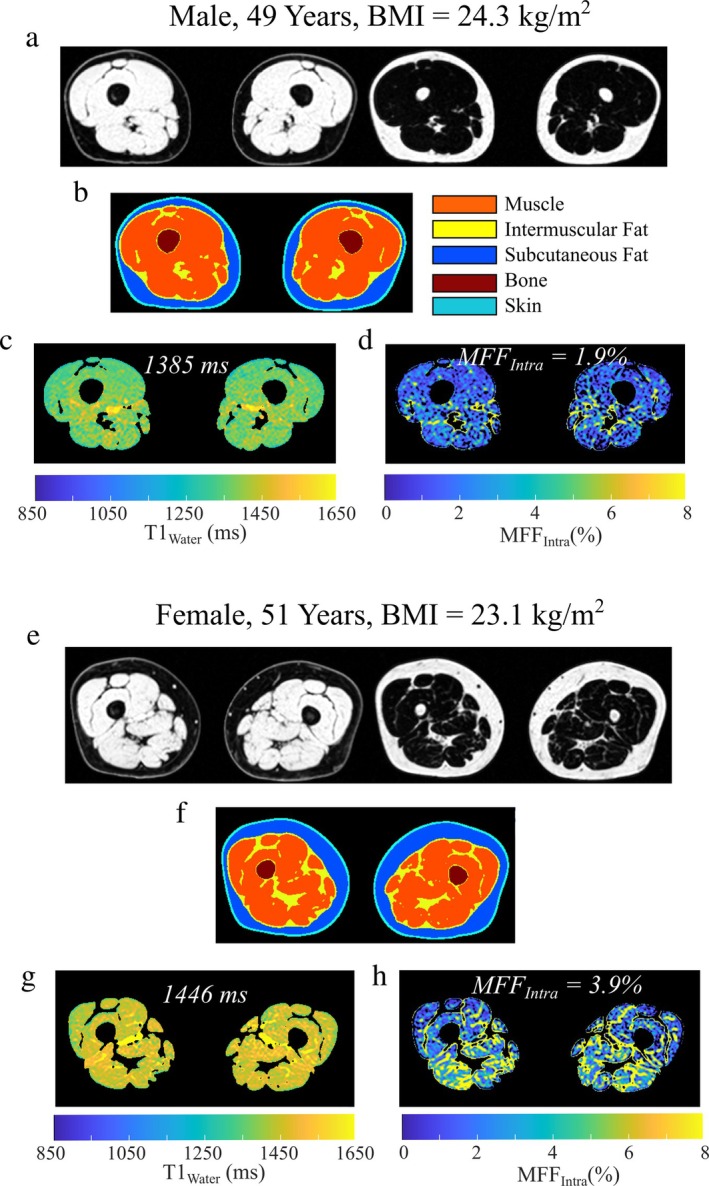
Illustrative calculated SR‐CSE images from a male (**a**–**d**) and female (**e**–**h**) participant: (a) Water and fat separated images (nonsaturation recovery), (b) machine learning segmentation, (c) T1_Water_ maps and (d) intramuscular fat fraction (MFF_Intra_) maps, with matched images in (e–h) for the female participant. T1_Water_ and MFF_Intra_ maps include pixels in the muscle segmentation region. One of five acquired slices is shown.

Fat and water separated images, machine learning segmentation, T1_Water_ and MFF_Intra_ maps from representative male and female participants illustrate typical image quality for the SR‐CSE approach (Fig. 3).

#### FAT QUANTIFICATION

**FIGURE 4:**
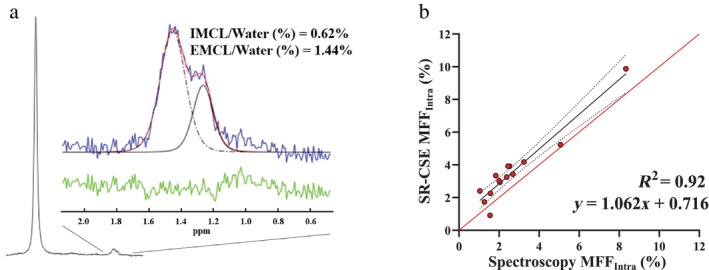
In vivo validation of SR‐CSE‐derived intramuscular fat fraction (MFF_Intra_). (**a**) Example of a single‐voxel ^1^H MRS spectra with fitting for intramyocellular (IMCL) and extramyocellular (EMCL) fat pools which together make up MFF_Intra_ = IMCL + EMCL (blue: acquired spectrum; green: residual after fitting). (**b**) Scatter plot showing the agreement between MFF_Intra_ derived from SR‐CSE and ^1^H spectroscopy.

Results for the in vivo spectroscopic validation of SR‐CSE‐derived MFF_Intra_ are shown in Fig. 4. A representative spectrum with best‐fit and residual curves identifies the intramyocellular and extramyocellular peaks that together make up the total MF_Intra_ pool (Fig. 4a). There was excellent agreement between SR‐CSE and ^1^H spectroscopy for MFF_Intra_ (*R* ^2^ = 0.92, slope coefficient of 1.06) with a positive bias of +1% for SR‐CSE with 95% limits of agreement of ~2%.

Comparison of fat volumes calculated with the SR‐CSE approach and conventional multi‐echo imaging used for CSE calculations yielded excellent agreement for comparison of MFV_Intra_, MFV_Inter_, MFF_Intra_, and MFF_Inter_ (*R* ^2^ = 0.8–0.99) in 41 participants (Fig. S4 in the Supplemental Material).

#### T1 QUANTIFICATION—PULSE SEQUENCE DEPENDENCE

**FIGURE 5:**
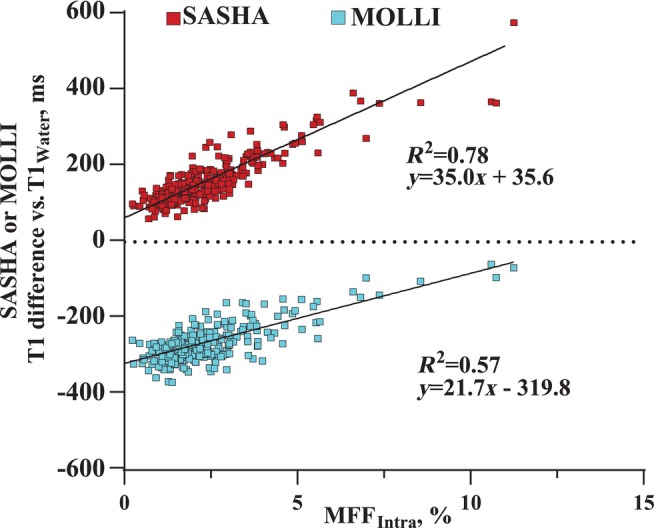
Influence of fat content on SASHA and MOLLI T1 bias in skeletal muscle. Comparison of T1 bias (SASHA or MOLLI T1 minus SR‐CSE T1_Water_) as a function of MFF_Intra_, measured with the SR‐CSE acquisitions, from a total of 277 regions of interest from nine healthy participants.

There was poor agreement between SR‐CSE muscle T1_Water_ with SASHA (*R* ^2^ = 0.18; 95% confidence interval [CI] of 243 msec) and MOLLI (*R* ^2^ = 0.21; 95% CI of 176 msec) T1 mapping methods, from a total of 277 regions of interest from nine participants. The wide range of disagreement is explained largely by T1 bias for MOLLI and SASHA associated with fat content, with a measured bias of +35 msec/1% MFF_Intra_ for SASHA and + 22 msec/1% MFF_Intra_ for MOLLI, as compared to SR‐CSE T1_Water_ (Fig. 5). Additionally, SASHA had a constant T1 bias of +36 msec and MOLLI had a constant T1 bias of −320 msec vs. SR‐CSE T1_Water_.

#### REPEATABILITY

SR‐CSE scan‐rescan repeatability (*n* = 20) was excellent for the two distinct muscle fat pools, MFF_Inter_ (CV = 0.3%) and MFF_Intra_ (CV = 2.6%), subcutaneous fat (CV = 0.82%) and muscle volumes (CV = 0.18%) and for T1_Water_ (CV = 0.08%) and R2* values (1.70%).

#### NORMATIVE VALUES FOR MUSCLE T1_WATER_ AND FAT COMPOSITION IN HEALTHY ADULTS AND THE EFFECTS OF SEX AND AGE

Demographic characteristics for study participants are outlined in Table 1. The mean age of the cohort was 47 ± 15 years (range: 18–76 years) with 63 males and 67 females, who were predominantly of Caucasian ethnicity. On average the cohort had a normal BMI, with 35% classified as overweight or obese (BMI ≥ 25.0 kg/m^2^).

**TABLE 1:** Participant Demographics

Characteristic	Total (*n* = 130)	Males (*n* = 63)	Females (*n* = 67)
Male, *n* (%)	63 (48%)	NA	NA
Age (years)	47 ± 15 (18–76)	47 ± 15 (18–75)	48 ± 16 (18–76)
Height (cm)	170.2 ± 9.0	176.6 ± 7.0	164.3 ± 6.1
Weight (kg)	71.7 ± 12.6	77.9 ± 11.3	65.8 ± 10.9
BMI (kg/m^2^)	24.7 ± 3.6	24.4 ± 4.1	24.9 ± 3.0
<18.5	2 (1%)	1 (2%)	1 (2%)
18.5–24.9	83 (64%)	36 (57%)	47 (70%)
25.0–29.9	32 (25%)	23 (36%)	9 (13%)
>30.0	13 (10%)	3 (5%)	10 (15%)
Ethnicity			
Caucasian	108 (83%)	51 (81%)	57 (85%)
Asian	15 (12%)	7 (11%)	8 (12%)
Indigenous	2 (1%)	1 (2%)	1 (2%)
African American	1 (1%)	1 (2%)	0 (0%)
Hispanic	1 (1%)	1 (2%)	0 (0%)
Other	3 (2%)	2 (3%)	1 (2%)
Lifestyle factors			
Previous smoker, *n* (%)	19 (15%)	8 (13%)	11 (16%)
Regular alcohol, *n* (%)	79 (61%)	37 (59%)	42 (64%)
Drinks/week^a^	1.3 (0–4.3)	1.0 (0.0–4.0)	2.0 (0.0–5.0)

Data are mean ± SD (range), or frequency (%).

BMI = body mass index.

^a^ Calculated only for those who reported regular alcohol consumption.

**FIGURE 6:**
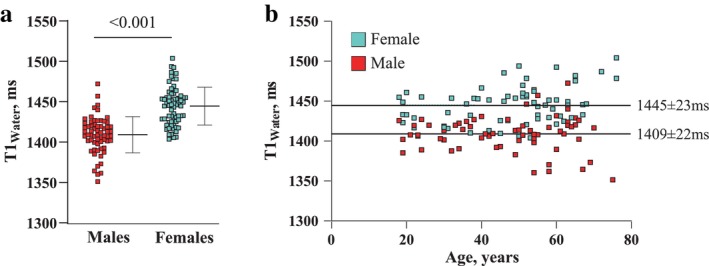
Association of muscle T1_Water_ with sex and age. (**a**) Individual T1_Water_ values (with overlay of mean and 95% CI) for males and females. (**b**) Scatter plot showing T1_Water_ values for males and females as a function of increasing age. Dotted line corresponds to sex‐specific mean values shown in (a).

The sex‐specific median, upper (95th percentile) and lower limits (5th percentile) for the T1 and thigh composition values for all participants are shown in Table 2. Sex‐specific values for T1_Water_ and the influence of sex and age on T1_Water_ are shown in Fig. 6. T1_Water_ was significantly higher in females (+36 msec), with no age‐dependence. The impact of sex and age on all measures of thigh composition were assessed using multivariable linear regression (Table 3). Overall, there was a significant impact of sex on most outcomes, such that females had lower absolute MFV_Intra_ and lower absolute volume and percentage of muscle tissue in the thigh, but higher muscle T1_Water_ and higher fat fractions, MFF_Total_ and MFF_Inter_, with a tendency for higher MFF_Intra_. Age was not significantly associated with T1_Water_ (*P* = 0.07), but was significantly and positively associated with increasing subcutaneous fat, as well as the total volumes and fractions of MF_Total_, MF_Inter_, and MF_Intra_. In contrast, increasing age was associated with lower volume and percentage of thigh muscle. Due to significant collinearity with fat volumes, BMI was not included in the multivariable model. However, comparing muscle composition and T1_Water_ between those with normal vs. overweight‐obese BMI (Table S5 in the Supplemental Material) showed that those with increased BMI had significantly higher volumes and fat fractions for all fat pools, lower muscle percentage and increased R2*, but no differences in absolute skeletal muscle volumes (males, *P* = 0.41; females, *P* = 0.51) or T1_Water_ values (males, *P* = 23; females, *P* = 0.28). We performed the same SR‐CSE acquisition in an individual with type 2 diabetes to explore the potential utility of T1_Water_. This individual had T1_Water_ values (1467 msec) ~1SD above the 95th percentile values for T1_Water_ for males in our healthy cohort (Fig. S6 in the Supplemental Material).

**TABLE 2:** Normative Muscle T1_Water_ and Fat Composition Values for Males and Females

Measure	Males			Females		
	5th Percentile	Mean ± SD	95th Percentile	5th Percentile	Mean ± SD	95th Percentile
Thigh						
SCF volume (mL)	84	288 ± 150	531	354	718 ± 307	1368
Muscle volume (mL)	1052	1306 ± 162	1604	715	983 ± 162	1248
Muscle percentage (%)	63.8	76.4 ± 7.6	89.5	35.8	54.8 ± 9.7	68.3
MFV_Total_ (mL)	60	127 ± 51	237	76	129 ± 43	220
MFV_Inter_ (mL)	37	96 ± 41	184	60	103 ± 37	187
MFV_Intra_ (mL)	15	31 ± 12	63	14	26 ± 9	45
MFF_Total_ (%)	3.9	8.8 ± 3.1	13.9	7.2	11.8 ± 4.2	19.3
MFF_Inter_ (%)	2.6	7.1 ± 2.9	12.4	5.9	10.4 ± 4.8	20.1
MFF_Intra_ (%)	1.1	2.3 ± 0.8	3.8	1.3	2.6 ± 1.0	4.7
Muscle T1_Water_ (msec)	1362	1409 ± 22	1445	1408	1445 ± 23	1490
Muscle R2* (msec)	39	42 ± 2	44	38	41 ± 2	45
Paraspinal muscle						
MFF_Intra_ (%)	1.3	2.7 ± 1.1	4.9	1.7	3.4 ± 1.7	6.7
Muscle T1_Water_ (msec)	1391	1422 ± 22	1470	1396	1428 ± 18	1458

SCF = subcutaneous fat; MFV_Total_ = total muscle fat volume; MFV_Inter_ = intermuscular fat volume; MFV_Intra_ = intramuscular fat volume; MFF_Total_ = total muscle fat fraction; MFF_Inter_ = intermuscular fat fraction; MFF_Intra_ = intramuscular fat fraction.

**TABLE 3:** Relationship Between Thigh Composition, Age, and Sex

Measure	*β* (SE)	95% CI	SD β	*P*	Adjusted *R* ^2^
SCF (mL)					
Intercept	164.5 (21.6)	121.7 to 207.2	–	<0.001	0.45
Age (years)	1.2 (0.4)	0.4 to 2.0	0.18	0.005	
Male sex	−127.7 (12.7)	−152.9 to −102.5	−0.65	<0.001	
Muscle (mL)					
Intercept	334.2 (13.9)	306.7 to 361.6	–	<0.001	0.53
Age (years)	−0.8 (0.3)	−1.3 to −0.2	−0.17	0.006	
Male sex	95.5 (8.2)	79.3 to 111.7	0.71	<0.001	
Muscle (%)					
Intercept	63.2 (2.5)	58.3 to 68.1	–	<0.001	0.64
Age (years)	−0.2 (0.1)	−0.3 to −0.1	−0.20	<0.001	
Male sex	21.5 (1.5)	18.6 to 24.4	0.78	<0.001	
MFV_Inter_ (mL)					
Intercept	19.8 (3.2)	13.4 to 26.3	–	<0.001	0.08
Age (years)	0.2 (0.1)	0.1 to 0.3	0.29	<0.001	
Male sex	−2.1 (1.9)	−5.9 to 1.6	−0.10	0.26	
MFV_Intra_ (mL)					
Intercept	3.11 (0.09)	1.44 to 4.78	–	<0.001	0.25
Age (years)	0.09 (0.02)	0.06 to 0.13	0.45	<0.001	
Male sex	1.63 (0.50)	0.64 to 2.61	0.25	0.001	
MFV_Total_ (mL)					
Intercept	23.0 (3.9)	15.3 to 30.6	–	<0.001	0.11
Age (years)	0.3 (0.1)	0.2 to 0.5	0.35	<0.001	
Male sex	−0.5 (2.3)	−5.0 to 4.0	−0.02	0.82	
MFF_Inter_ (%)					
Intercept	5.84 (1.11)	3.64 to 8.04	–	<0.001	0.26
Age (years)	0.10 (0.02)	0.06 to 0.14	0.35	<0.001	
Male sex	−3.28 (0.66)	−4.59 to −1.98	−0.38	<0.001	
MFF_Intra_ (%)					
Intercept	0.97 (0.22)	0.54 to 1.41	–	<0.001	0.34
Age (years)	0.03 (0.01)	0.03 to 0.04	0.58	<0.001	
Male sex	−0.24 (0.13)	−0.49 to 0.02	−0.13	0.07	
MFF_Total_ (%)					
Intercept	6.71 (1.00)	4.73 to 8.67	–	<0.001	0.30
Age (years)	0.11 (0.02)	0.07 to 0.15	0.41	<0.001	
Male sex	−2.91 (0.59)	−4.07 to −1.74	−0.37	<0.001	
Muscle T1_Water_ (msec)					
Intercept	1433.3 (6.8)	1419.9 to 1446.7	–	<0.001	0.38
Age (years)	0.2 (0.1)	0.0 to 0.5	0.13	0.07	
Male sex	−35.3 (4.0)	−43.2 to −27.4	−0.61	<0.001	
Muscle R2* (msec)					
Intercept	39.00 (0.50)	38.01 to 39.98	–	<0.001	0.15
Age (years)	0.05 (0.01)	0.03 to 0.06	0.38	<0.001	
Male sex	0.41 (0.29)	−0.17 to 0.99	0.11	0.17	

SCF = subcutaneous fat; MFV_Inter_ = intermuscular fat volume; MFV_Intra_ = intramuscular fat volume; MFV_Total_ = total muscle fat volume; MFF_Inter_ = intermuscular fat fraction; MFF_Intra_ = intramuscular fat fraction; MFF_Total_ = total muscle fat fraction.

#### RELATIONSHIP BETWEEN THIGH AND PARASPINAL MUSCLE T1_WATER_ AND MFF_INTRA_

**FIGURE 7:**
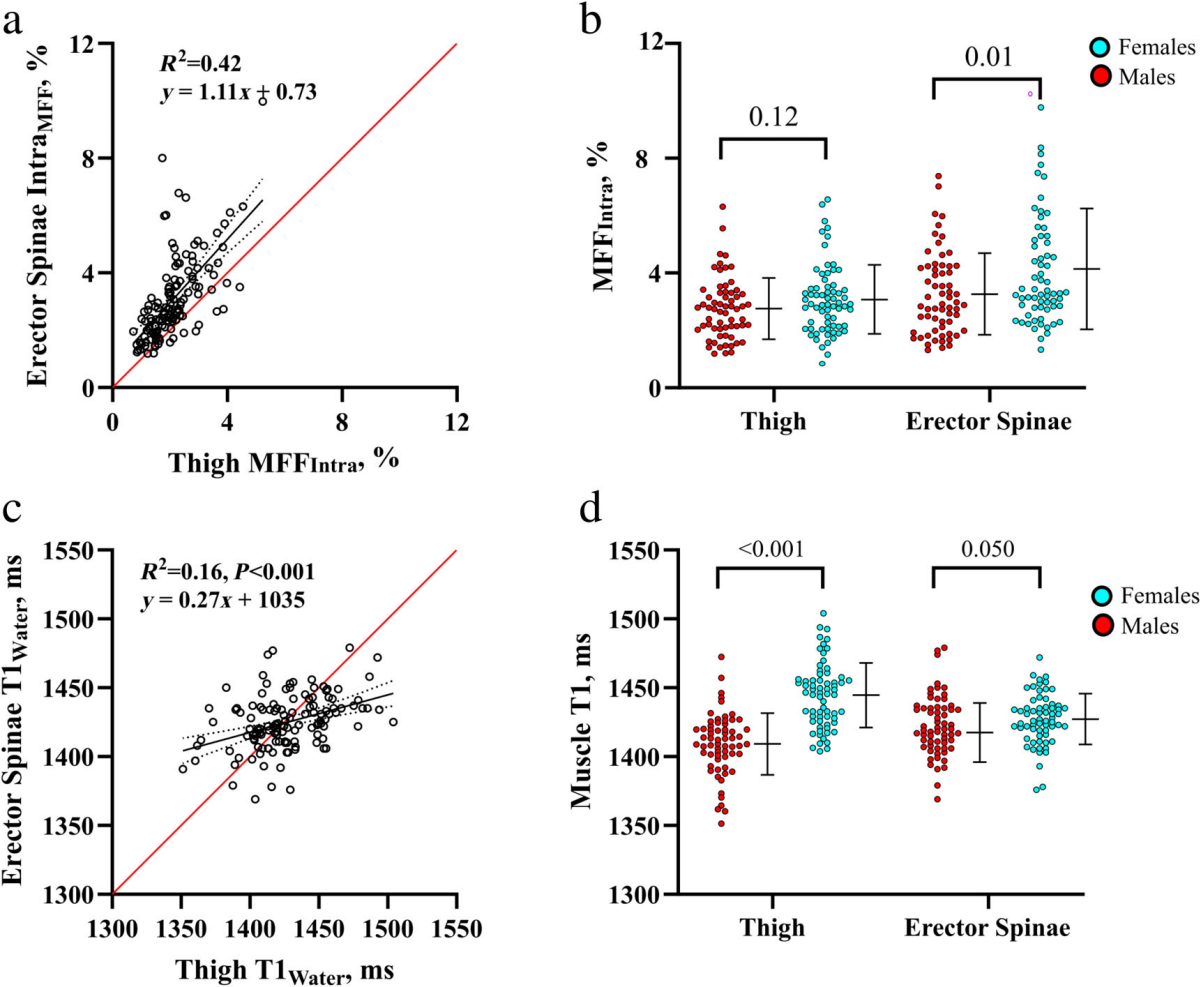
Agreement for muscle T1_Water_ and MFF_Intra_ in the thigh and paraspinal muscles. (**a**) Scatter plot showing agreement between values for MFF_Intra_ and (**c**) T1_Water_ in the erector spinae vs. mid‐thigh; Solid black line and adjacent dotted lines represent mean regression line and corresponding 95% confidence interval, respectively, while the solid red‐line represents the line of identity. (**b**) Values for MFF_Intra_ and (**d**) muscle T1_Water_ grouped according to sex for each muscle region (thigh or erector spinae). Overlay and error bars represent mean and 95% confidence interval, respectively, with *P*‐values for comparison of males vs. females is shown above.

There was good agreement for MFF_Intra_ between the thigh and paraspinal muscle groups (*R* ^2^ = 0.42), although with a small positive bias in the paraspinal muscles (+0.73%; Fig. 7a). Paraspinal MFF_Intra_ was also significantly higher in females (+0.70%; Fig 7b). There were no significant differences in T1_Water_ between thigh and paraspinal muscles (*P* = 0.39), but only modest agreement in paired analysis of T1_Water_ values (*R* ^2^ = 0.16; Fig. 7c). The sex‐difference in T1_Water_ values of the thigh was also seen in the paraspinal muscles, although it was of a lesser magnitude as compared to thigh muscle values, +6 msec vs. + 36 msec for females vs. males, respectively (Fig. 7d).

## Discussion

A SR‐CSE approach, using a combination of saturation recovery preparation and chemical shift encoded methods,^17^, ^24^ provided repeatable quantification of water‐specific T1 values in skeletal muscle (T1_Water_) and good agreement with phantoms. A fat‐mediated T1 bias was shown for conventional T1 mapping methods, even in healthy individuals with relatively low intramuscular fat content. Skeletal muscle T1_water_ values were higher in females but with no age dependence. The SR‐CSE approach provided simultaneous quantification of muscle and fat composition; the different fat pools (subcutaneous, intermuscular, intramuscular) and skeletal muscle volume had distinct dependencies on age and sex, for the thigh muscle. User‐independent segmentation of muscle, subcutaneous fat, MF_Inter_, and bone using a custom nnU‐Net machine learning approach (detailed in the Supplemental Material) was successful in all participants, likely contributing to the excellent repeatability of muscle and fat volumes and T1_Water_ values. The SR‐CSE approach offers joint assessment of intrinsically registered fat and T1_Water_ values, providing quantitative measures of complimentary disease processes. The insensitivity of the specific implementation of the SR‐CSE approach in the current study to factors such as B_1_^+^ and B_0_ inhomogeneity^17^ may also contribute to good reproducibility.

### Normative T1 Values in Skeletal Muscle

Myocardial T1 assessment is now a fairly routine approach for the assessment of fibrosis, edema and inflammation, which are also core elements of skeletal muscle disorders including acute muscle injury, neuromuscular disorders, disuse, aging (sarcopenia), and obesity. However, there is no consensus on normative skeletal muscle T1 values, their age and sex dependence or the impact of MF_Intra_ on muscle T1 values. In the current study, in 130 healthy individuals aged 18–76 years, the mean T1_Water_ values for thigh muscles were 1409 ± 22 msec (males) and 1445 ± 23 msec (females) using the SR‐CSE approach. A similar, but shorter, breath‐hold duration SR‐CSE approach^17^ applied to the paraspinal muscles in this same cohort yielded similar T1_Water_ values, suggestive of uniformity of muscle T1 values across muscle types in health. Previous studies at 3 T reported muscle T1 values of 1420 msec at the level of the knee using a Look‐Locker method^29^ and 1412 msec using an inversion recovery approach in excised muscle tissue samples,^30^ similar to values in the current study. However, significantly lower thigh muscle T1 values at 2.89 T were measured using an inversion‐recovery radial acquisition approach, but still with higher T1 values in females, 1242 ± 21 msec, as compared to males, 1221 ± 21 msec,^12^ in agreement with the current study. Muscle T1 values measured using the DESPOT‐1 approach ranged from 1240 to 1370 msec in 47 healthy individuals at 2.89 T.^31^ They reported no significant sex dependence, but showed a strong association between T1 and fat fraction, suggesting significant fat‐related T1 bias. However, some studies noted above were completed at 3 T, as compared to 2.89 T in the current study (and other previous studies using a Siemens ‘3 T’ Prisma or Tim Trio), so direct comparison between studies should be interpreted against the potential influence from the field strength dependence of T1. Recent studies also acknowledge the need to remove the confounding effects of fat.^16^, ^22^, ^23^, ^24^ An MR fingerprinting approach that included fat‐water separation reported mean muscle T1_Water_ values of 1199 msec at 2.89 T.^14^ However, concurrent phantom validation of this approach showed a systematic underestimation of T1_Water_ by 230 msec, suggestive of unbiased values in the range of 1400 msec, similar to values reported in the current study.

The significantly higher muscle T1_Water_ values in females independent of muscle fat content in our relatively large cohort of 130 participants, is consistent with T1 mapping studies in the heart^32^ and liver.^24^ This emphasizes the importance of considering sex when interpreting muscle T1_Water_.

### Intramuscular Fat and Bias in T1 Mapping

This study validated the accuracy of the SR‐CSE approach for the assessment of T1_Water_ values in the presence of fat. Using water‐specific methods will avoid fat‐modulated T1 bias which is advantageous given the co‐existence of fatty replacement and fibrosis in muscle disorders and the well‐documented confounding influence of fat on T1 times in mixed water and fat systems.^9^, ^16^, ^17^, ^18^ Our study showed that commonly used SASHA and MOLLI T1 mapping methods had poor agreement with SR‐CSE T1_Water_ values in skeletal muscle, driven primarily by a fat‐modulated T1 bias. In particular, the measured T1 bias on order of 20–30 msec per 1% intramuscular fat content suggest that even small MFF_Intra_ values on the order of 2%–3% would shift a normal T1_Water_ value into the abnormal range (i.e., ≥2 standard deviations above the expected mean values), independent of changes in muscle edema or fibrosis. The magnitude of the fat‐related T1 bias will depend on the specific T1 mapping approach used and even the particular pulse sequence parameters and thus these current results, for SASHA and MOLLI pulse sequences, cannot be generalized to other T1 mapping approaches.

### Muscle and Fat Volumes

Healthy aging was associated with a statistically significant, but relatively modest reduction in muscle volume, and accumulation of absolute fat volumes (subcutaneous, MF_Inter_, MF_Intra_).^4^, ^33^ This meant that muscle fat fractions (muscle fat as a percentage of muscle volume), MFF_Inter_ and MFF_Intra_, increased to a greater extent with age than absolute volumetric changes owing to both a loss of muscle volume and an accumulation of fat with increasing age. However, in our healthy cohort, the effects of age on muscle composition were substantially smaller than the effects of sex, highlighting the sex‐dependence of muscle composition even in healthy individuals. In agreement with previous studies using MRI or computed tomography, female participants had higher fat fractions and lower muscle volumes.^4^, ^33^, ^34^ The MFF_Intra_ was also consistent between thigh and paraspinal muscle groups, raising the possibility that MFF_Intra_ may largely be determined by more systemic processes (at least in healthy individuals) than muscle‐group specific factors.

Spectroscopic validation of MFF_Intra_ illustrated the accuracy of these measurements even for low values (~2% MFF_Intra_). Our SR‐CSE‐derived MF_Inter_ and MF_Intra_ values had excellent agreement with a conventional CSE approach for PDFF assessment, which will enable comparison of our reported values to previous or future studies using the widely available CSE approach. However, correction of the intrinsic T1‐weighting of the conventional CSE data was necessary to avoid overestimating fat content due to the shorter T1 values of fat (~370 msec) compared to muscle water (~1400 msec). By comparison, the SR‐CSE approach used in the current study utilized spin‐density weighing in the nonsaturation recovery images using a ramped flip angle approach to avoid the need for T1 correction of fat fractions.^17^

The majority of imaging‐based studies of skeletal muscle‐fat focus on the larger and more visible intermuscular deposits. In particular, T1‐weighted or qualitative dual‐echo Dixon approaches have utilized a binary masking approach to identify pixels as fat or not.^11^, ^35^, ^36^, ^37^ These methods are limited by the need to define arbitrary signal intensity thresholds to identify fat pixels, and a strong dependence on spatial resolution. The CSE approaches offer quantification of fat content in every pixel (i.e., PDFF), which enables the evaluation of MF_Intra_. Accurate and reproducible differentiation of MF_Intra_ from MF_Inter_ may be important as these are distinct fat depots, and may have differential associations with the pathophysiology and outcomes of different disease processes.^2^, ^3^ Alternatively, this approach also allows targeting total muscle fat content (intermuscular plus intramuscular) eliminating the need for segmentation of the distinct fat pools, a process that may depend on highly variable factors such as spatial resolution.

### Limitations

Studies were limited to 2.89 T and thus future studies are required to determine field‐strength specific normative skeletal muscle T1_Water_ values. Generalizability of T1 and muscle fat content finding is limited due localized coverage including the mid‐thigh and erector spinae muscle groups. Differentiation of MF_Inter_ and MF_Intra_ is confounded by partial‐volume effects given the limited spatial resolution of MRI, and relative volumes will likely depend on the acquired resolution. The MF_Intra_ pool includes a mixture of intramyocellular and extramyocellular lipids of unknown proportions, with potentially distinct pathophysiological associations that cannot be effectively differentiated by the approach used in this study. Our study focused on T1_Water_ values in healthy, predominantly Caucasian adults, so we cannot determine how T1_Water_ values are impacted by muscle disorders or ethnicity. Interestingly, when we evaluated T1_Water_ in a single individual with type 2 diabetes—which is a condition that has been associated with both increased MFF_Intra_ and skeletal muscle collagen content^38^—we found T1_Water_ values that were ~1 SD above the 95th percentile values for T1_Water_ for males in our healthy cohort. Nevertheless, future clinical studies are necessary to determine the implications of elevated skeletal muscle T1 values in a broad range of relevant patient populations (neuromuscular disorders, myopathies, orthopedics, metabolic disease, sarcopenia); ideally with some link to histopathology (such as biopsy) for mechanistic insight, or linkage to clinical endpoints to understand its clinical relevance.

## Conclusion

This study demonstrated the accuracy and repeatability of the SR‐CSE multiparametric pulse sequence for simultaneous quantification of skeletal muscle T1_Water_ and composition, including MF_Intra_, MF_Inter_, subcutaneous fat and muscle volumes, in healthy individuals, including normative values and sex‐dependence of T1_Water_ values. We have also demonstrated the significant bias in T1 values associated with MF_Intra_ content using convention T1 mapping methods. Overall, the SR‐CSE pulse sequence may provide useful insights into pathological skeletal muscle changes seen with aging, cancer, and neuromuscular, orthopedic, and metabolic diseases.

## Supporting information


**Data S1.** Supporting Information.
