# Supplementary material for: Reference Values for Water‐Specific T1, Intermuscular and Intramuscular Fat Content in Skeletal Muscle at 2.89 T
Source: J Magn Reson Imaging. 2025 Jan 24;62(1):146–59. doi: 10.1002/jmri.29718 (PMC12179366; doi:10.1002/jmri.29718)
Supplement: Supplementary file 1 — Data S1. Supporting Information. [file JMRI-62-146-s001.docx]

**S1. Phantom Studies for Validation of the SR-CSE Pulse Sequence**

*S1.1 Phantom Studies: T1 Validation*

Twelve NiCL_2_-agarose phantoms with a wide range of T1 and T2 values were used for evaluation of T1 accuracy of the SR-CSE sequence without fat content.  Reference T1 values were measured using a saturation-recovery gradient-echo pulse sequence (TE = 3.6 ms, TR = 10 seconds, 30° flip angle, 300 mm field of view, 128×64 matrix, TS = (30ms, 100 ms, 200ms, 400ms, 600ms, 800ms, 1000ms, 1500ms, 3000ms, 5000ms, 8000ms).  Each phantom was constructed in a 50 ml plastic sample tube and all samples were placed in a bath of gadolinium doped water (T1~300 ms) for simultaneous sample imaging.

*S1.2 Phantom Studies: T1_Water_ and PDFF Validation*

A second set of five phantoms with a range of targeted fat content [0%, 5%, 10%, 20%, 30%], constructed using a standardized approach(1), were used to evaluate accuracy of T1_Water_ in the presence of fat with simultaneous PDFF quantification using the SR-CSE sequence. The T1 of the water solutions in the mixed fat-water phantoms was targeted to approximately 1000 ms using 0.18 mM Gadavist (Bayer HealthCare Pharmaceuticals, Montville, NJ). Each fat phantom was constructed in a 50 ml plastic sample tube and all samples were placed in a bath of gadolinium doped water (T1~1300 ms) for simultaneous sample imaging.  Reference values for T1_Water_ and PDFF in the mixed fat-water phantoms were measured using a custom saturation-recovery single-voxel spectroscopy pulse sequence. The spectroscopy sequence was based on the STEAM approach (TE = 15 ms, TM (mixing time) = 10 ms, TR = 10 seconds, 1300 Hz/pixel bandwidth, 1 cm × 1 cm × 1 cm voxel volume), using the same optimized saturation pulse as the SR-CSE sequence, and saturation recovery times of 300 ms in steps of 100 ms to 1000 ms and a non-saturation recovery acquisition.  PDFF was evaluated using the area, *A*, under the water peak and sum of the fat peaks, *A_fat_* / (*A_wate_*_r_ + *A_fat_*), from the non-saturation recovery spectra. The fat and water areas were corrected for T2 signal loss using STEAM acquisitions with TE = 15 ms, 30 ms and 50 ms.


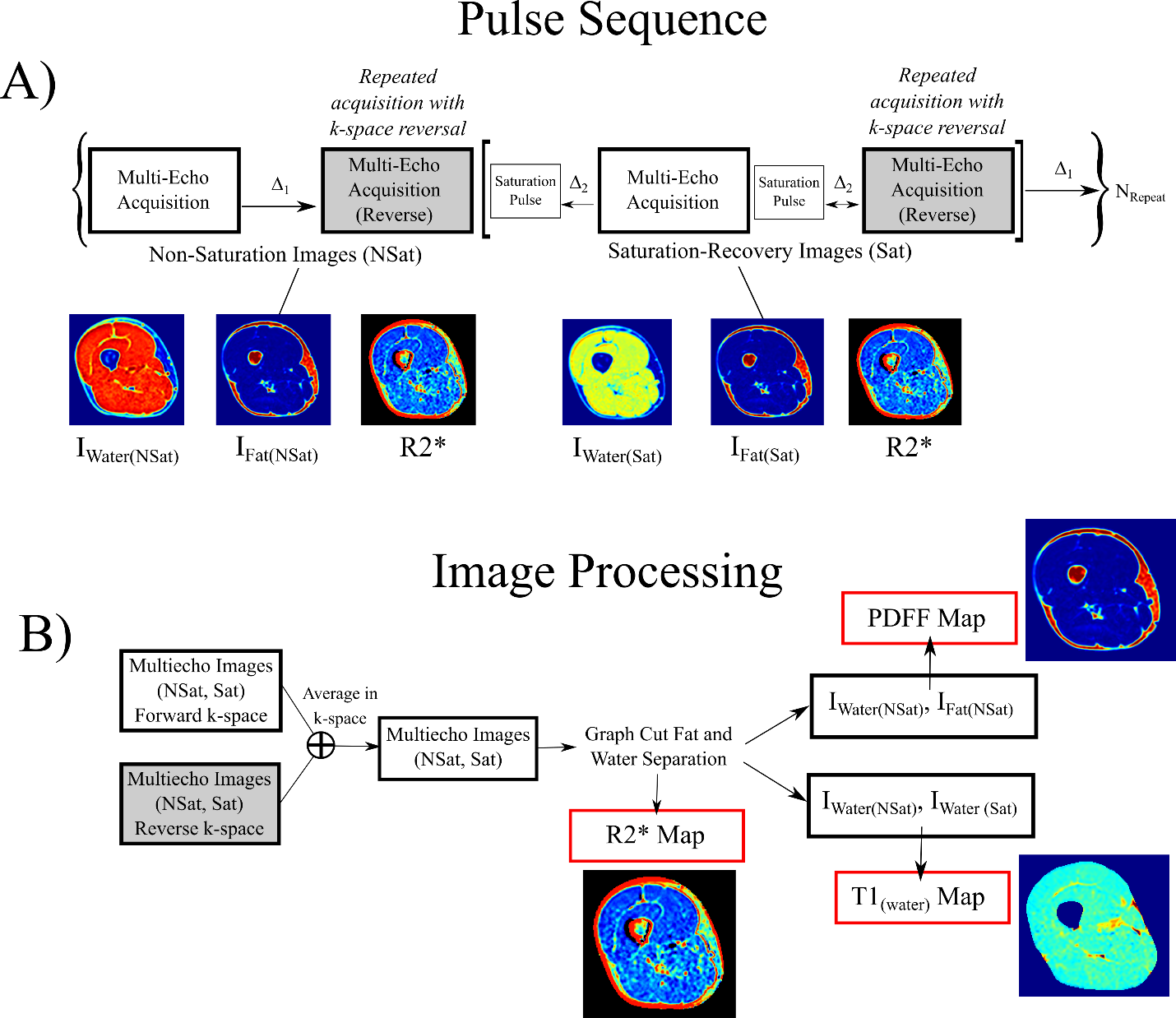


**Supporting Figure S2 – SR-CSE pulse sequence and image processing**. The details of the SR-CSE pulse sequence have previously been published and validated.^1^ **A)** Single shot multi-echo gradient-echo acquisitions are repeated with saturation preparation (Sat) and without (NSat). Images acquisitions are repeated with a reversal of k-space in the phase-encoding direction. The thigh SR-CSE acquisitions in the current study employed three repetitions (N_Repeat_ = 3) to implement echo shifting in each repetition, to increment the echo times. **B)** Forward and reverse k-space data are averaged prior to image reconstruction.  CSE-MRI processing of multi-echo images yields water and fat separated images, with and without saturation preparation, I_Water(NSat)_, I_Fat(NSat)_, I_Water(Sat)_ and I_Fat(Sat)_.  Fat fraction (PDFF) maps were calculated from I_Water(NSat)_ and I_Fat(NSat)_, and water-specific T1 maps (T1_(Water)_) were calculated from I_Water(NSat)_ and I_Water(Sat)_.  R2* maps were reconstructed as part of the CSE-MRI image processing. Δ_1_ = 8 seconds and Δ_2_ = 0 seconds.

1. Thompson RB, Chow K, Mager D, Pagano JJ, Grenier J. Simultaneous proton density fat-fraction and R2∗ imaging with water-specific T1 mapping (PROFIT1): application in liver. Magn Reson Med. 2021;85(1):223-238.

**S3. Development and Validation of Machine Learning Segmentation Thigh Muscle and Fat**

The fat and water separated images were used to segment the thigh images (both thighs) into five targeted regions including subcutaneous fat, muscle, intermuscular fat, bone and skin. Given its impressive performance for numerous of biom9edical segmentation tasks, nnU-Net(1) was used to train a deep learning framework to these targeted regions. nnU-Net is a self-configuring convolutional neural network that assesses dataset characteristics including imaging modality, intensity distribution, median image shape, and distribution of voxel spacing, using a U-Net architecture(2). By default, nnU-Net is trained using a combination Dice similarity and cross-entropy loss function with stochastic gradient descent optimization. nnU-Net was implemented with Python 3.8 on a Linux operating system and was trained on our datasets using an NVIDIA V100 GPU on Compute Canada.

Prior to images segmentation, fat and water separated images were spatially normalized to remove coil shading. Ground truth mask generation first targeted the muscle region. Briefly, pixels that were predominantly water were first automatically identified in the water-separated images using an intensity threshold from which seed pixels were randomly selected in the muscle region. An iterative region growing approach(3) was subsequently used to add neighboring pixels to grow the muscle region based on signal intensity being within a percentage difference (typically 5%) from seed pixels, which was repeated until the number of pixels added in one iteration was less than a pre-defined value, typically 20 new pixels. Next, a user-independent active contour approach was used to trace the outer boundary of the muscle with no user interaction, which was used to identify the boundary of the muscle and subcutaneous fat pool. The region growing algorithm was next applied to the subcutaneous region (with exclusion of the muscle region identified via the active contour), using the fat-separated images. Skin was identified using a signal intensity threshold on water-separated images, using the edge of the subcutaneous fat region to limit the search region to the outer edge. A point in the bone marrow region was selected by the user on the fat-separated images and the region growing algorithm was used to identify the full bone, including an image threshold to identify the cortical bone region surrounding the marrow. Finally, the region within the active contour, that was outside of the identified muscle and bone regions, was assigned as intermuscular fat.

A training dataset included 25 thigh image sets, each with 5 slices, for a total of 125 two-dimensional fat and water separated image pairs, including both thighs. A separate validation dataset included 167 participants, for a total of 835 slices. Training and validation cases were acquired as part of separate study of post-COVID multi-organ tissue damage(4).

The performance of the machine learning model was evaluated using the Dice similarity coefficient, with values of 0.98 for subcutaneous fat, 0.93 for skin, 0.93 for muscle, 0.99 for intermuscular fat and 0.98 for bone, with a typical case shown in Fig. S2. All SR-CSE cases in the current study were segmented with the validated machine learning model, including the 41 conventional CSE cases.


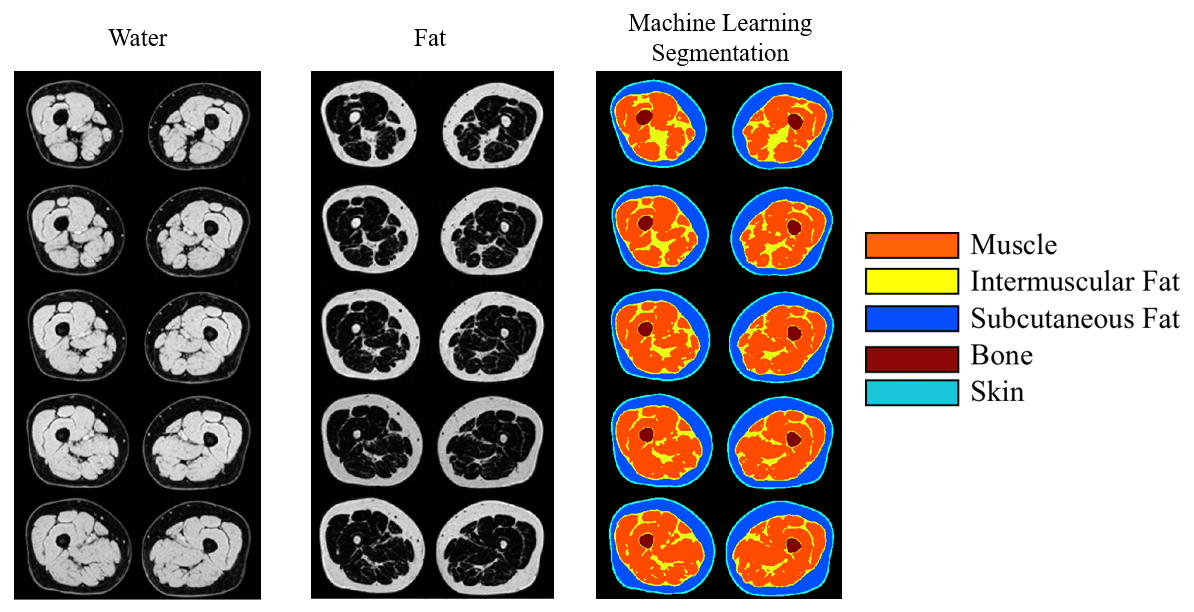
***Supporting Figure S3* *– Machine Learning Segmentation of Thigh Images***. A typical thigh muscle image dataset from the SR-CSE approach (5 slices) showing water and fat separated images and the corresponding machine learning segmentation.

Intermuscular and intramuscular fat volumes, MFV_Inter_ and MFV_Intra_, were measured as the sum of MFF_Inter_ and MFF_Intra_ values (fat fractions), respectively, from the corresponding segmented regions multiplied by the pixel volume. The total volume of muscle fat (MFV_Total_) was defined as the sum of MFV_Inter_ and MFV_Intra_ (in ml) in the muscle and MF_Inter_ regions, for which no intermuscular segmentation is required (i.e. total fat from the muscle and MF_Inter_ regions) and was expressed relative to the total volume of pixels in the muscle region to calculate total muscle fat fraction (MFF_Total_). Muscle volume was divided by the sum of the total tissue volume (sum of muscle, subcutaneous, MF_Inter_ and MF_Intra_) to derive the muscle percentage.

**References**

1) Isensee F, Jaeger PF, A KSA, Petersen J, Maier-Hein KH. nnU-Net: a self-configuring method for deep learning-based biomedical image segmentation. *Nat Methods*. 2021;18(2):203-211.

2) Ronneberger O, Fischer P, Brox T. U-Net: Convolutional Networks for Biomedical Image Segmentation. Published online May 18, 2015.

3) Becker A. Region Growing (2D/3D) in C. Published December 2, 2017. Accessed August 21, 2023. <https://www.mathworks.com/matlabcentral/fileexchange/63317-region-growing-2d-3d-in-c>.

4) Paterson DI, White JA, Beaulieu C, et al. Rationale and design of the multi organ inflammation with serial testing study: a comprehensive assessment of functional and structural abnormalities in patients with recovered COVID-19. *Front Med (Lausanne)*. 2024;11:1392169.


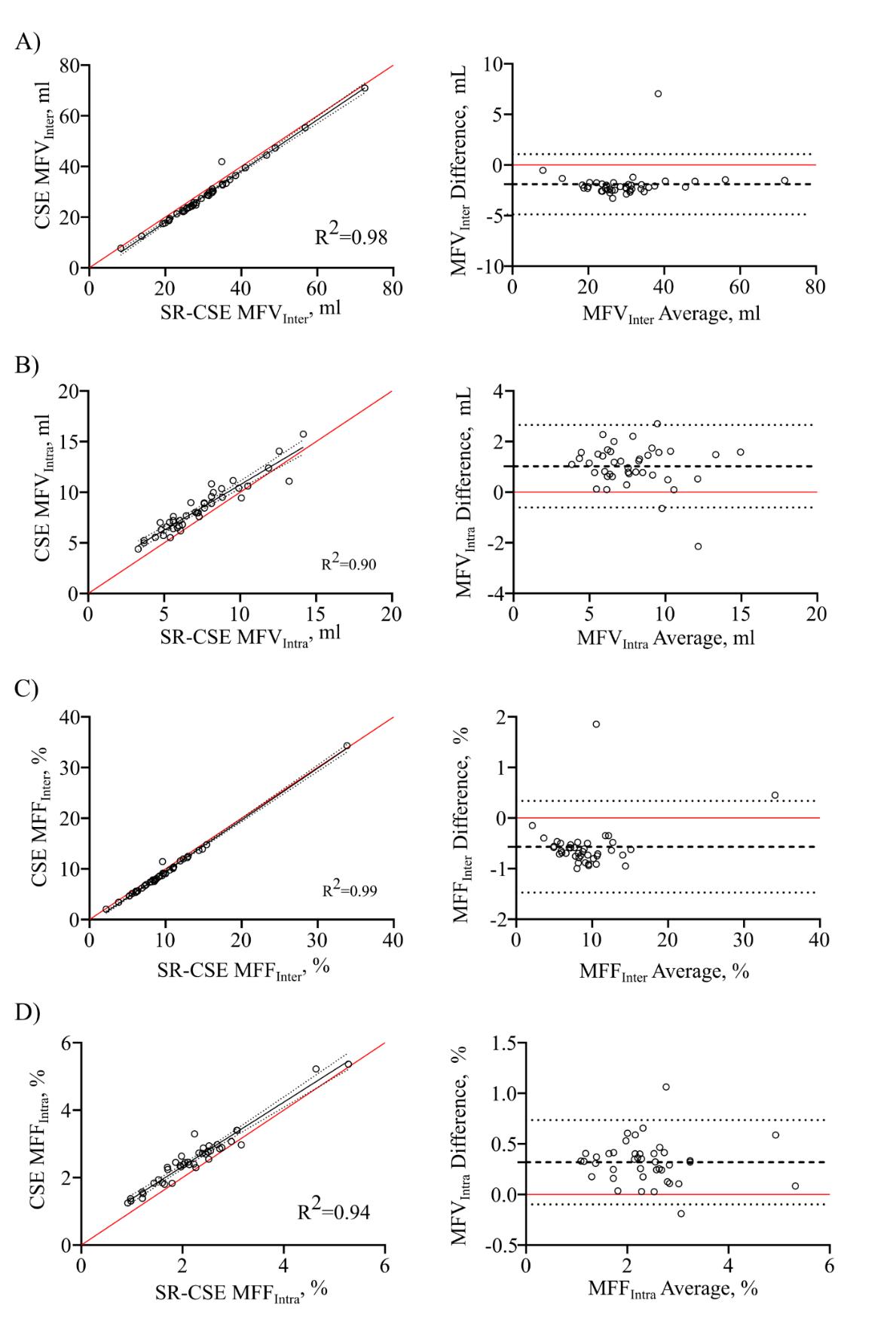


**Supporting Figure S4 – Comparison of intermuscular (Inter_MF_) and intramuscular (Intra_MF_) fat volumes and fat fractions for conventional chemical-shift encoded MRI and the multiparametric SR-CSE approach.** Correlation and Bland and Altman plots are shown for, **A)** intramuscular fat volume (Inter_MFV_), **B)** intramuscular fat volume (Inter_MFV_), **C)** intermuscular fat volume (Inter_MFV_) **D)** intramuscular fat volume (Intra_MFV_). Data is from 41 healthy participants (from the main study healthy cohort).

| **Supporting Table S5.** Effect of BMI on thigh and paraspinal muscle composition in healthy males and females. | | | | | | |
| --- | --- | --- | --- | --- | --- | --- |
| **Measure** | **Males** | | | **Females** | | |
|  | ***BMI <25 kg/m^2^*** | ***BMI ≥25 kg/m^2^*** | ***P-value*** | ***BMI <25 kg/m^2^*** | ***BMI ≥25 kg/m^2^*** | ***P-value*** |
| ***Thigh*** |  |  |  |  |  |  |
| SCF Volume, mL | 244±109 | 351±179 | 0.031 | 588±164 | 1046±342 | <0.001 |
| Muscle Volume, mL | 1292±172 | 1326±147 | 0.41 | 975±142 | 1004±207 | 0.51 |
| Muscle % | 79.2±7.1 | 72.5±6.4 | <0.001 | 58.4±7.2 | 45.7±9.5 | <0.001 |
| MFV_Total_, mL | 98±24 | 168±52 | <0.001 | 112±26 | 173±48 | <0.001 |
| MFV_Inter_, mL | 73±21 | 127±42 | <0.001 | 89±21 | 140±44 | <0.001 |
| MFV_Intra_, mL | 25±6 | 41±13 | <0.001 | 23±8 | 33±9 | <0.001 |
| MFF_Total_, % | 7.2±2.0 | 11.2±2.9 | <0.001 | 10.4±2.6 | 15.2±5.5 | <0.001 |
| MFF_Inter_, % | 5.7±2.0 | 9.2±2.8 | <0.001 | 9.0±2.6 | 14.2±6.9 | <0.001 |
| MFF_Intra_, % | 1.9±0.5 | 2.9±0.9 | <0.001 | 2.3±0.8 | 3.2±0.9 | <0.001 |
| Muscle T1_Water_, ms | 1403±19 | 1417±25 | 0.23 | 1441±24 | 1453±21 | 0.28 |
| Muscle R2*, ms | 41.0±1.7 | 42.3±1.6 | 0.001 | 40.7±1.7 | 42.4±1.8 | <0.001 |
| ***Paraspinal Muscle*** |  |  |  |  |  |  |
| MFF_Intra_, % | 2.3±1.0 | 3.4±0.9 | <0.001 | 3.0±1.4 | 4.6±1.9 | <0.001 |
| Muscle T1_Water_, ms | 1420±22 | 1426±20 | 0.24 | 1426±19 | 1430±18 | 0.57 |
| Data are mean±SD. Abbreviations: BMI, body mass index; SCF, subcutaneous fat; MFV_Total_, total muscle fat volume; MFV_Inter_, intermuscular fat volume; MFV_Intra_, intramuscular fat volume; MFF_Total_, total muscle fat fraction; MFF_Inter_, intermuscular fat fraction; MFF_Intra_, intramuscular fat fraction. | | | | | | |


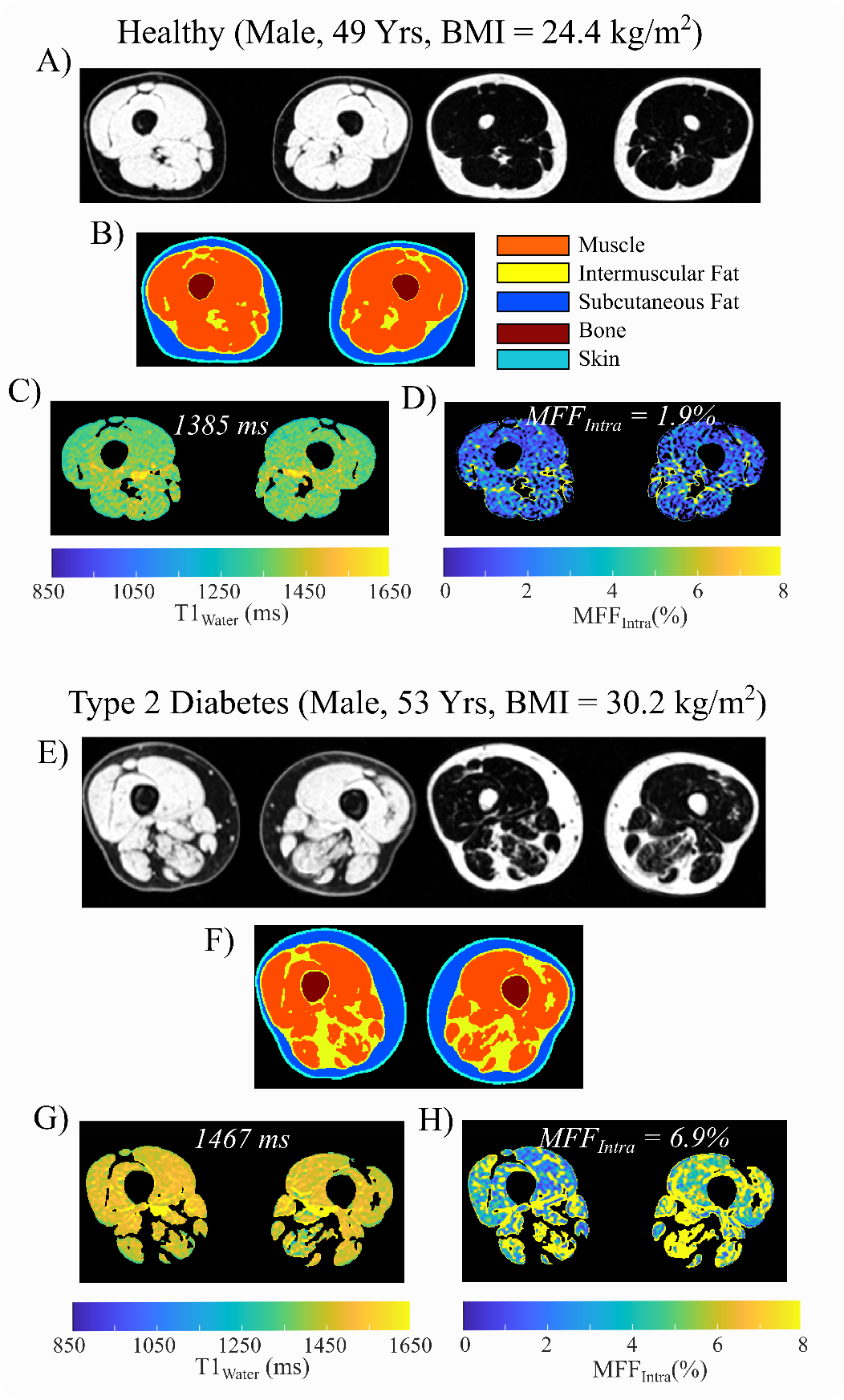


**Supporting Figure S6. Illustrative calculated SR-CSE images from a healthy, normal weight male (A-D) and an obese male with Type 2 Diabetes (E-H): A)** Water and fat separated images (non-saturation recovery), **B)** machine learning segmentation, **C)** T1_Water_ maps and **D)** intramuscular fat fraction (MFF_Intra_) maps, with matched images in **E) – H)** for the male with Type 2 Diabetes. One in five acquired slices is shown.
